# Incentives and organ donation: what’s (really) legal in Canada?

**DOI:** 10.1186/2054-3581-1-7

**Published:** 2014-05-29

**Authors:** Timothy Caulfield, Erin Nelson, Brice Goldfeldt, Scott Klarenbach

**Affiliations:** Faculty of Law and School of Public Health, Health Law Institute, University of Alberta, 116 St and 85 Avenue, Edmonton, AB T6G 2R3 Canada; School of Public Health, 3-300 Edmonton Clinic Health Academy, University of Alberta, 11405 – 87 Avenue, Edmonton, AB T6G 1C9 Canada; Department of Medicine, Faculty of Medicine and Dentistry, University of Alberta, 116 St and 85 Avenue, Edmonton, AB T6G 2R3 Canada

**Keywords:** Organ donation, Legal, Ethics, Incentives

## Abstract

**Purpose of review:**

To date, there has been little analysis of the degree to which emerging incentive initiatives are permissible under Canadian law. The purpose of this review is to examine the relevant law – including legislation and case law – in order to clarify the legality of existing proposed incentive schemes.

**Sources of information:**

Legislation and case law.

**Findings:**

Organ donation is governed by provincial legislation that, in general, bans the exchange of any “benefit” or any form of “valuable consideration” in return for an organ. As such, these laws are tremendously restrictive and could have significant implications for emerging and proposed procurement policy.

**Implications:**

Given the need for innovative, ethically appropriate policies to increase donation rates, we suggest that the time is right to rethink the potentially restrictive nature of Canada’s organ donation laws.

## Résumé

**But de l’article:**

À ce jour, il y a eu peu d’analyse légale des incitatifs pour le don d’organes au Canada. Le but de cet article est d’examiner les lois relatives au don d’organes afin de déterminer la légalité de différents incitatifs.

**Sources d’informations:**

Les textes de loi et la jurisprudence.

**Résultats:**

Le don d’organes relève de compétences provinciales, lesquelles, de manière générale, interdisent au donneur de recevoir tout avantage ou compensation en échange de son organe. Ces lois sont extrêmement restrictives et peuvent donc avoir des conséquences significatives sur de futures politiques de don et de prélèvement d’organes. Ceci étant dit, il est cependant difficile d’émettre des prédictions définitives sur les interprétations possibles de la loi par les tribunaux.

**Implications:**

Compte tenu la nécessité d’augmenter le don d’organes via des politiques innovatrices et éthiquement acceptables, nous croyons qu’il est opportun de revoir les lois canadiennes sur le don d’organes afin d’éliminer les aspects restrictifs de ces dernières.

## What was known before

There seems be an assumption that the existing ban on valuable consideration in the context of organ donation applies only to direct financial payments in exchange for organs.

## What this adds

It is clear that existing legislative bans on the use of, for example, “valuable consideration” have broad application and may apply to a range of existing and proposed incentive initiatives.

## Introduction

The need to increase rates of organ donation has received growing attention in both the healthcare policy literature [1–5] and in the popular press [6]. Canada’s donation rates fall well below many other OECD nations [7, 8] and as the demand continues to grow, the pressure to find ways to improve the supply of organs intensifies. The perennial organ shortage has led to the introduction of, and speculation about, a number of policy reforms [9], including the use of financial incentive schemes for both living and deceased donation (Table 1).

**Table 1:** **Example incentive initiatives**

Incentive	Valuable consideration?
**Priority as a potential recipient in exchange for donating**	**Likely**. Donor has potential to receive a substantial benefit in return for registration.
Potential donors who indicate a willingness to donate or who register to donate post mortem are placed on a priority list to receive an organ should they need one [3, 40].	
**Donor recognition events**	**Likely**. While the provisions of gifts to donors could be viewed as a form of reward that would, technically, be a form “valuable consideration”, it seems too insignificant to be considered a policy concern (more a symbolic gesture).
Events hosted by organ procurement organizations in order to recognize living and deceased donors [41–43].	
**Paired exchange programs**	**Likely**. If participating in the program increases your chance of your intended recipient receiving an organ, it could be viewed as a benefit and, as a result, valuable consideration (hence the modification of the US law to allow such programs).
These programs facilitate kidney donations by pairing those in need of a kidney with a willing but non-compatible donor with other such pairs.	
**Reimbursement of out-of-pocket expenses and lost wages**	**Possibly**. The degree to which these programs could constitute a “benefit” would likely need to be evaluated on a case-by-case basis and may depend, inter alia, on the degree to which payment is truly for quantifiable costs. This would be equally true for policies that seek to provide money as a way to compensate for pain and suffering.
Donors often incur expenses during the organ donation process. Some programs reimburse donors for these expenses, including travel, food and accommodation costs [3, 4, 12–14].	
**Funeral expenses**	**Yes**. The payment of costs of this nature would clearly infringe existing legislation as it is the provision of a benefit in exchange for donation.
A system whereby donors have all or a portion of funeral expenses paid for or reimbursed [1–3, 25, 26].	
**Tax credit**	**Yes**. Even though not a direct payment, still an obvious financial benefit.
A system whereby donors are granted a tax credit by either the federal or provincial government for donation organs [3, 44].	
**Cash incentives**	**Yes**.
Payment of money for organs [1, 2, 4, 33].	

Canada’s organ donation system is premised on altruism [10] and Canadian lawmakers have gone to great lengths to ensure that the system is structured to prevent commercial exchange of organs. The provincial laws that form the primary framework for our organ donation system – and which formalize the altruistic foundations of the system – have been in place for years [11]. But as the clinical and policymaking communities struggle to meet the growing demand for organs, a range of initiatives have been considered [3, 12–14] or rolled out that provide incentives or, as they are often framed, seek to remove disincentives.

Despite this policy trend, there seems to have been little analysis of whether (and, if so, what types of) incentives are permissible under Canadian law. In this paper we review the relevant law on organ donation and ask whether there is scope for lawful incentive strategies. We also suggest that, in light of existing and proposed initiatives and the clear demand for organs, a more flexible approach to regulation – one that has room for evidence-based and ethically sound innovation – is warranted. As we will see, many of the proposed and existing initiatives may conflict with current law. But our goal is *not* to discourage the introduction of such policies. On the contrary, we feel the time is right to rethink the potentially restrictive nature of Canada’s organ donation laws.

## Review

### The Law

In Canada, the provinces and territories are responsible for regulating organ and tissue transplantation. Legislation in each province and territory sets norms regarding organ donation, and each such statute includes what amounts to a ban on the buying and selling of organs. Although there is some variation in the exact wording of the relevant provisions, every province has a prohibition that, taken literally, is tremendously broad in scope. Seven of the thirteen statutes state that “No person shall buy, sell or otherwise deal in, directly or indirectly, for a valuable consideration, any tissue for a transplant, or any body or parts other than blood or a blood constituent, for therapeutic purposes, medical education or scientific research” [15–21]. In Québec, the Civil Code provides that “[t]he alienation by a person of a part or product of his body shall be gratuitous…” [22]. In other provinces, such as Alberta, the legislation prohibits the exchange of organs for “any reward or benefit” [10].

In law, “valuable consideration” is expansively defined; it is understood to mean any detriment incurred by one party to an agreement, or any benefit received by the other party [23, 24]. Consideration is much more than a mere exchange of money for goods or services. A classic example that highlights the breadth of the legal conception of valuable consideration arises from an English case, *Chappell & Co v Nestlé*[25], wherein the House of Lords held that chocolate bar wrappers could be viewed as consideration. Likewise, the concept of “reward or benefit” is extremely far-reaching. In referring to “valuable consideration” and “reward or benefit”, the statutes arguably prohibit any exchange that could be viewed as creating an incentive to donate organs.

If an individual or organization were to challenge the legal legitimacy of a particular incentive program, the court would be required to interpret the provision that prohibits dealing for an organ “for a valuable consideration”. Based on the broad approach to the meaning of consideration in the case law, the court would very likely be persuaded that the legislature chose the language of “valuable consideration” deliberately, and with a view to including much more than an outright purchase of organs. In other words, if the legislature had intended only to prohibit the exchange of cash for organs or tissues, then the provision could have been drafted much more narrowly. The choice of the language of consideration suggests that all programs that can be viewed as providing a benefit to the donor in exchange for an organ could be interpreted as being contrary to the spirit of the legislative prohibition, and therefore illegal.

It is important to note that our interpretation of “valuable consideration” is based on the common law of contract. Canadian organ donation legislation makes it an offence to contravene of the law. This implies that the law may be interpreted more narrowly, in keeping with the “strict construction” approach used in the context of penal legislation, where uncertainty or ambiguity is resolved in favour of the accused person [26]. Still, as noted above, valuable consideration should be viewed as much more than money. It can be almost anything that serves as a reason, motive or inducement for entering into an agreement.

### Incentive policies

As noted, numerous incentive mechanisms have been either implemented or proposed [See Table 1], including the provision of a tax credit to living donors and the payment of some or all of a deceased donor’s funeral expenses [1–3, 27, 28]. Both of these types of incentives are clearly “benefits” to the donor or donor’s family, and therefore infringe existing provincial legislation.

Providing full or partial reimbursement of a living donor’s expenses has also been suggested as a way to increase donation frequency, and this approach has been adopted by a number of provinces [13, 14]. For example, Ontario has the “Program for Reimbursing Expenses of Living Organ Donors” which allows donors to claim up to $5,500 for out of pocket expenses [13]. Québec has a similar program [14]. While these programs are hardly generous – indeed, research has shown that the personal financial costs of organ donation often far exceed the $5,500 limit [12]– they may nevertheless infringe the ban on the exchange of valuable consideration in return for an organ. Many policy documents seek to draw a clear distinction between incentives and the repaying expenses (or removing “disincentives”) [29, 30]. But from a legal perspective, the line is not always clear. Simply declaring a payment to be a reimbursement or the removal of a “disincentive” does not, in the eyes of the law, necessarily mean it could not be construed as a benefit or valuable consideration. As we have seen in the context of other debates, such as with surrogacy arrangements [31, 32], there is much debate about what is a legitimate expense and when reimbursement crosses the line to “payment”. Interestingly, Manitoba is the one province that has amended its legislation to specifically provide for an “exception as to expenses”, thus highlighting the degree to which the provision of expenses could be problematic (if not, why create the clarifying exception?). The Manitoba law states that nothing in the act “prohibits reimbursement” to the donor, recipient of an organ or family members [21].

A more complex type of incentive to donate organs is what has been referred to as a “paired exchange program”, wherein kidney donations are facilitated by pairing those in need of a kidney with a willing but non-compatible donor with other such individuals. These programs exist throughout the world, including in Canada [33] and, if perceived as a way of increasing the chance of the donor’s intended recipient receiving an organ, can be viewed as providing an incentive. In the US, the law that prohibits the exchange of “valuable consideration” for organs was amended to explicitly provide that the ban does not apply to “human organ paired donation” [34]. The fact of this amendment highlights the reality that, but for the amendment, the practice would be caught by the broad prohibition against incentives.

## Conclusion

There are significant distinctions – both real and semantic – among various types of incentive schemes aimed at increasing organ supply. At one end of the continuum are initiatives that seem relatively benign, including removal of disincentives and reimbursement of a donor’s actual expenses. At the other end are schemes that more clearly infringe the spirit of the existing laws, including the outright purchase of organs, or payment of compensation to the organ provider in return for the “donation”. Other proposals seem to sit nearer the centre, in that although they do not appear to involve the exchange of cash for organs, they still have an obvious pecuniary element (examples include payment of funeral expenses and the provision of a tax credit).

The aim of all of these proposed and existing incentives policies is to increase the supply of organs, thereby reducing wait times and improving the likelihood that those in need will eventually receive an organ [35]. Yet although most agree on the ultimate goal, there is an ongoing debate in the transplant community around the ethical acceptability of incentives for organ donation [1, 28]. The perspective favouring incentives appears to be stronger in public opinion: recent research has found that 71% of the Canadian public would support the use of some form of financial incentive to increase donation rates. A surprising 45% support the use of a regulated system whereby monetary payment for organs [2] is made to donors by the regulator.

Despite the increasing focus on such schemes, there remains much uncertainty and imprecision in popular representations of the law, which often reduce the legal rule prohibiting the exchange of consideration to its most basic, stating simply that “selling organs is illegal in Canada” [6]. Some organizations (such as the Kidney Foundation of Canada [29]) have adopted policies that reject “economic incentives” for organ donation, but nevertheless support “reimbursement” of out of pocket expenses for living donors, implying that there is an obvious difference between the two practices. And other commentators have made similar arguments [28]. But, as is clear from the above, the current legal framework makes few distinctions. The prohibition on incentives is a relatively broad ban that reaches well beyond just the “selling of organs” and is relevant to all policies that involve something that could be conceived as “valuable consideration.”

And, despite the apparently definitive legislative statements about what is proscribed and what is, by implication, permitted, there is little to guide us, as far as government policy and legislative debates, in interpreting the relevant statutes. It seems likely that the intent was for the prohibitions to be broadly interpreted. The objective is, rightly or not [27], to preserve the altruistic nature of donation. During the enactment of the most recent version of Alberta’s statute, for example, comments made by lawmakers emphasized the need to ensure that the giving of an organ “must be as a gift, and we must see it as a gift” [36]. Similar pronouncements can be found in earlier legislative debates as well. During a 1983 legislative debate in Ontario it was suggested that “We cannot change this gift relationship with respect to human organ transplants” [37]. In Alberta in 1973, it was noted that the then proposed law “prohibits the sale of tissue … and it is a serious offence to sell that tissue” [38].

If we are correct in our broad interpretation of the prohibitions, Canadian policy is tremendously restrictive in the context of incentives; so much so that most proposed and existing incentive programs seem to have the potential to violate the letter of the law. We want to be very clear: we raise this point not to insist that these programs and efforts be abandoned. Rather, our view is that Canadian lawmakers need to take a close look at the justifications for the sweeping prohibitions found in the legislation. Based on emerging policy initiatives and public sentiment [2], it is fair to say that existing law may be out of step with current views and the needs of the transplant community. Laws prohibiting the payment of consideration for organs were put in place at a time when organ transplantation was in its infancy. Transplantation is now a routine and cost-effective treatment option in numerous disease contexts. And, as with all new medical technologies, there has been a significant shift in public sentiment around the ethical acceptability of solid organ transplantation since the first successful kidney transplants in the 1950s [39].

In view of these factors, we urge a careful and critical review of organ donation laws in all Canadian jurisdictions, with a view to reconsidering the breadth of the legislative restrictions around organ donation. Changing legislation is not an easy task, but we can and should start to consider the best and most efficient way to toward policy reform. We recognize that the use of incentives should be just one of a range of policy strategies to increase the supply of organs. But Canada needs to have laws that can accommodate innovative and ethically acceptable approaches to increasing the supply of organs. There is a vast difference between permitting incentives like payment of some or all of a deceased donor’s funeral expenses or paired organ exchanges and allowing the purchase and sale of organs. Even to the extent that Canadians wish to maintain an organ donation system that is premised on altruism (a concept that is itself open to a great deal of interpretation), we think most would be prepared to say that incentives such as those we describe here are, if not ideal, at least preferable to the status quo, in which many patients die waiting for a life-saving organ transplant.
